# Pleiotropic Biological Effects of Dietary Phenolic Compounds and their Metabolites on Energy Metabolism, Inflammation and Aging

**DOI:** 10.3390/molecules25030596

**Published:** 2020-01-29

**Authors:** María del Carmen Villegas-Aguilar, Álvaro Fernández-Ochoa, María de la Luz Cádiz-Gurrea, Sandra Pimentel-Moral, Jesús Lozano-Sánchez, David Arráez-Román, Antonio Segura-Carretero

**Affiliations:** 1 Department of Analytical Chemistry, University of Granada, 18071 Granada, Spain; marivillegas@ugr.es (M.d.C.V.-A.); alvaroferochoa@ugr.es (Á.F.-O.); spimentel@ugr.es (S.P.-M.); ansegura@ugr.es (A.S.-C.); 2 Research and Development of Functional Food Centre (CIDAF), 18016 Granada, Spain; jesusls@ugr.es; 3 Department of Food Science and Nutrition, University of Granada, 18071 Granada, Spain

**Keywords:** pleiotropic, phenolic compounds, chronic disorders, metabolites, bioactive compounds, oxidation, inflammation, aging

## Abstract

Dietary phenolic compounds are considered as bioactive compounds that have effects in different chronic disorders related to oxidative stress, inflammation process, or aging. These compounds, coming from a wide range of natural sources, have shown a pleiotropic behavior on key proteins that act as regulators. In this sense, this review aims to compile information on the effect exerted by the phenolic compounds and their metabolites on the main metabolic pathways involved in energy metabolism, inflammatory response, aging and their relationship with the biological properties reported in high prevalence chronic diseases. Numerous in vitro and in vivo studies have demonstrated their pleiotropic molecular mechanisms of action and these findings raise the possibility that phenolic compounds have a wide variety of roles in different targets.

## 1. Introduction

Bioactive compounds are substances present as natural sources that have health benefits beyond the basic nutritional value of the product. By consensus, bioactive compounds have been defined as essential and non-essential compounds that are present in nature showing beneficial effects on human health [1]. Among the great variety of bioactive compounds, phenolic compounds stand out for the numerous amounts of compounds described with great bioactive potential and structural diversity. These compounds are secondary metabolites naturally synthesized in plants and present more than 8000 chemical structures [2].

As mentioned, phenolics have a wide physiological activity in humans, such as an antioxidant [3], antimicrobial [4], anti-allergenic, cardioprotective, antiatherogenic, anti-inflammatory [5,6,7,8], and anticancer activities [9]. Due to this diverse and potential bioactivity, phenolic compounds have become functional compounds of great interest to the scientific community [10]. However, when phenolic compounds are ingested, they are usually metabolized suffering various modifications in certain chemical groups by means of glucuronidation, methylation, or sulfation reactions, among others, which can alter the biological activity. Besides that, they can be catabolized by the colonic microflora, which may drastically affect the absorption of these molecules through the gut barrier [11]. For this reason, it is necessary to take into account these metabolic reactions as well as their bioactivity when evaluating the bioactivity of phenolic compounds.

Phenolic compounds have shown a beneficial role against chronic diseases mainly related to metabolic stress, inflammation, and aging. In the literature, extracts and isolated compounds from plants have been reported to act on the main metabolic pathways involved in energy metabolism, inflammatory response, and aging.

In these pathways, there are specific key proteins that act as regulators. The main regulators of energy metabolism are the AMP-protein kinase (AMPK) [12] and the mammalian target of rapamycin (mTOR) [13]. In the case of inflammatory response and aging, the nuclear factor-erythroid 2 p45-related factor 2 (Nrf2) and sirtuins are the main regulatory elements, respectively [14,15]. However, these routes do not act in isolation, otherwise they are connected to each other. In addition, results obtained by in vitro and in vivo models support that plant bioactive metabolites exert pleiotropic effects further than only free radical scavenging capacity [15,16,17,18,19]. They modulate multiple metabolic pathways through a variety of molecular targets, probably due to its molecular promiscuity and character diversity acquired through evolution [15].

In this context, the aim of this review is to gather information on the effects exerted by the phenolic compounds from plant sources and their metabolites in the main metabolic pathways involved in energy metabolism, inflammatory response, aging, and their relationship with the main chronic diseases with high prevalence. Specifically, *Silybum marianum, Lippia citriodora, Hibiscus sabdariffa, Theobroma cacao,* and *Olea europaea* have been selected due to the fact that the main families of phenolic compounds in the diet are fully represented in them.

A literature search of PubMed/MEDLINE using search terms ‘*Silybum marianum*’ ‘*Lippia citriodora*’ ‘*Hibiscus sabdariffa*’ ‘*Theobroma cacao*’ ‘*Olea europaea*’ AND ‘energy metabolism’ ‘inflammation’ ‘aging’, alone or in combination, was done up to 11 December 2019. Papers from the last decade were prioritized, and experimental studies that include the effect of named matrices on named processes were included both in vitro and in vivo in humans and in animal models.

## 2. Effect of Bioactive Compounds on Energy Metabolism

Energy metabolism is the processes that involve food intake, transforming the food to release energy and storing the excess through complex metabolic pathways within the cell. The deregulation of energy homeostasis has been related to numerous chronic diseases, such as cancer, inflammation, obesity, diabetes, etc. [20]. In this sense, it has been shown that AMPK, which is a highly conserved serine/threonine protein kinase formed by a heterotrimer with an α-catalytic subunit, a scaffolding β-subunit, and a regulatory γ-subunit, presenting each subunit multiple isoforms [12], is an important regulator of this cellular energy homeostasis [21].

AMPK is activated allosterically in cellular conditions with high concentration of AMP (ischemia, hypoxia, exercise, metabolic waste, and nutrient starvation) and is inhibited when the ratio ATP:AMP is high (nutrient-rich conditions) [22]. AMPK is also activated either by phosphorylation of Thr172 in the AMPKα by liver kinase B1 (LKB1) [23] or by Ca^2+^/calmodulin-dependent protein kinase β (CaMKK β) in response to elevated intracellular Ca^2+^ concentrations, independently of the energetic state of the cell [24].

AMPK takes part in the regulation of carbohydrate and lipid metabolism, and its activation resulting in the inhibition of ATP-consuming anabolic pathways, including fatty acid (FA) synthesis, hepatic gluconeogenesis, cholesterol, and isoprenoid synthesis, and mTOR-mediated protein translation. AMPK activation also stimulates ATP production through the increase of FA oxidation, muscle glucose transport, caloric intake and mitochondrial biogenesis [12,21]. In addition to the AMPK, there are other proteins involved in modulation of insulin effects on lipid metabolism as protein kinase B (Akt/PKB) and protein kinase C (PKC)-ζ [25].

Adipogenic transcription factors as PPARγ and C/EBP-α induce gene expression changes characteristic of mature adipocytes and remain elevated for the life of the cells [26]. These factors positively regulate each other’s expression since PPARγ can promote adipogenesis in C/EBPα-deficient cells [27]. Adipogenesis is the process by which undifferentiated precursor cells differentiate into fat cells and occurs in different stages. First, the growth-arrested preadipocytes initiate mitotic clonal expansion (MCE), this allows reentry into the cell cycle for another two rounds of division. After the MCE, the adipocyte differentiation is divided into the early, intermediate, and late stages. In the early stage, C/EBPβ and C/EBPδ increase, producing changes in the expression of PPARγ and C/EBPα that increase their levels until the beginning of the late stage when their levels start to lower gradually. Finally, the late stage is also regulated by PPARγ and C/EBPα which have a synergistic effect [28]. On the other hand, there are several proteins that act in the regulation of the lipogenesis, such as fatty acid synthase (FAS) and sterol regulatory element-binding proteins (SREBPs). FAS is a multi-enzyme protein that catalyzes fatty acid synthesis. The expression of this protein is regulated by multiple transcription factors. For example, SREBPs is one of these transcription factors, which also regulates the expression of proteins that participate in processes such as synthesis of cholesterol, triacylglycerols, and phospholipids [29].

It is also known that AMPK can inhibit the de novo synthesis of FAs either by direct phosphorylation and inhibition of the enzymes acetyl-CoA carboxylase (ACC) [30] and 3-hydroxy-3-methyl-glutaryl (HMG)-CoA reductase [31] or by the inhibition of the SREBP-1c [29].

Regarding energy stress the AMPK can be activated by phosphorylation, then the mTOR pathway is negatively regulated by directly phosphorylating the regulatory-associated protein of mammalian target of rapamycin (raptor) and tuberous sclerosis complex protein-2 [12,13]. In the plasma membrane, numerous receptors such as G protein-coupled receptor (GPCR); insulin-like growth factor receptor (IGF-R) and insulin receptor (IR) catch the signal that chemokines and growth factors wield on the cell that have a positive effect on mTOR through PI3K/Akt, since a serine/threonine protein kinase is activated by Akt [32]. Moreover, mTOR interacts with certain proteins to form two distinct complexes named mTOR complex 1 (mTORC1) and 2 (mTORC2). mTORC1 responds to energy, stress, amino acids, oxygen, and growth factors and is sensitive to rapamycin. It promotes cell growth by inducing anabolic processes and inhibiting catabolic processes. In the case of mTORC2, it responds to growth factors and regulates cell survival and metabolism, as well as the cytoskeleton, and this is not sensitive to rapamycin [33].

In summary, mTOR participates in the regulation of several processes such as protein synthesis and degradation, cell survival, longevity, and proliferation. Also, mTOR signaling pathway plays a fundamental part in the regulation of adipose tissue functions such as lipid metabolism, adipogenesis, and thermogenesis [34]. The deregulation or the chronic activation of mTOR may contribute to the pathogenesis of chronic diseases, such as obesity, non-alcoholic fatty liver disease, tuberous sclerosis and Alzheimer’s disease [32]. In this sense, it has been discussed the mechanisms by which polyphenols can delay the molecular pathogenesis of oxidative stress via inhibition of mTOR-signaling pathways. Besides the main mentioned pathways, the mammalian sirtuin protein family (comprising SIRT1-SIRT7) are enzymes with an important role in the control of the metabolic status. Sirtuins are nicotinamide adenine dinucleotide dependent (NAD) enzymes that regulate the action of target transcription factors and other enzymes by deacetylation at acyl-lysine residues or ADP-ribosylation that allows the control of the organismal health-span and life-span [35]. In addition, several studies have shown the benefits of phenolic compounds in neurodegenerative and metabolic disorders by activating certain sirtuins [36].

Table 1 shows different types of trials related to the effect of the selected plants on the main metabolic pathways related to energy metabolism.

### 2.1. Silybum Marianum

The action of several compounds or the entire extract of *S. marianum* have been evaluated by in vitro models in different cell lines such as human liver cells, T lymphocytes and HK2 cells.

Lovelace et al. (2015) studied the role of silymarin in the cellular metabolism of both human liver and T cells (Huh7.5.1 human hepatoma and Jurkat T cells). Silymarin is a flavonoid extract that contains silibinin, isosilybin, silydianin, and silychristin, obtained from the plant *S. marianum*. This study showed that nontoxic doses of silymarin first induced energy stress responses and then, the cells respond to these stresses with reparative and adaptive changes instead of dying. Moreover, silymarin modulates metabolic pathways, like AMPK, resulting in the suppression of mTOR and inflammatory signaling [13]. This action can be divided into two phases. The first phase consists in the induction of the stress response when the activation of AMPK signaling. In addition, the induction of DNA-damage-inducible transcript 4 mRNA and protein expression result in the inhibition of mTOR signaling translating to endoplasmic reticulum stress induction. The second phase results in the inhibition of the inflammation because the activation of AMPK signaling inhibits the nuclear factor-κB (NF-κB) signaling. This fact shows that the AMPK presents a major role in transducing the signal from silymarin-induced stress to anti-inflammatory effects. The effect of silymarin in the inflammation has also been explained for the Forkhead box O3 inhibition [58], a key conduit between cell metabolism, growth, and inflammation, and which is associated with suppression of many inflammatory genes such as C-X-C motif chemokine 10, whose expression has been associated with many autoimmune diseases [59].

As mentioned earlier, mTOR has several regulatory routes, for example PI3K/Akt/mTOR signaling route. Gharagozloo et al. (2012) investigated the effect of silymarin on the cell cycle and the PI3K/Akt/mTOR signaling pathway of activated T lymphocytes. They showed that silymarin inhibited cell proliferation through the suppression of the mTOR signaling pathway and also through G1 cell cycle arrest in human activated T lymphocytes in vitro [37].

The effect of some compounds of *S. marianum* have also been evaluated in both sirtuin 3 (SIRT3) knockout mice and diabetic Wistar rats. In this case, silybin, the major pharmacologically active compound of the *S. marianum* fruit, has also showed the ability to protect against cisplatin-induced acute kidney injury and tubular cell apoptosis both in vitro (HK2 cells) and in vivo (SIRT3 knockout mice) by improving mitochondrial function through the elevation of SIRT3 expression [54]. Besides, it has also been reported that silymarin extracts have an effect on sirtuin 1 (SIRT1). The administration of silymarin causes a positive regulation of SIRT1 mRNA expression, and this overexpression is related to a decrease in lipid concentrations, a higher glycogen content and a negative regulation of the SREBP-1c gene in the diabetic Wistar rats liver. This is a very important achievement in the field of diabetes research, since diabetes causes a significant increase in fasting blood sugar and in total cholesterol and triglycerides in the liver [60].

As well as silymarin induces a stress response and suppresses inflammation, there are other natural phytochemicals derived from plants with this capacity, for example, curcumin and epigallocatechin have demonstrated the same effects in AMPK pathways [61,62].

### 2.2. Lippia Citriodora

There are several studies that prove the effect of *L. citriodora* extract or some of its compounds on energy metabolism, for example, this effect has been studied in hypertrophic adipocytes, PC12 cells, and colorectal cancer cells.

Verbascoside is the most abundant phenylpropanoid glycoside compound in *L. citriodora*. This plant is known to possess multiple biological activities including anti-inflammatory, antioxidant, and anticancer as well as anti-obesity effects with AMPK activation [50]. For example, the incubation of high glucose-induced hypertrophic adipocytes with extract of *L. citriodora* or verbascoside produces a change in mRNA expression and protein levels of PPARα, a major regulator of lipid metabolism, FAS, lipogenic-related gene, and the central metabolic sensor AMPK. The result of the incubation is an increase in PPARα, a decrease in FAS and a significant activation of AMPK [38].

29 compounds from *L. citriodora* obtained by a HPLC semi-preparative purification method were tested in 3T3-L1 hypertrophic adipocyte model to prove their influence in the activation of AMPK. This activation was measured by immunofluorescence microscopy, resulting in that flavonoids, phenylpropanoids and iridoids, mainly luteolin-7-diglucoronide, verbascoside, and gardeoside, respectively, were the compounds with greater ability to activate AMPK. It was also observed that several compounds showed a synergic behavior, which provided a greater capacity of activation of the AMPK [39]. Similarly, Olivares-Vicente et al. (2019) also observed this synergic effect being verbascoside, luteolin-7-diglucuronide and loganic acid the compounds with greatest capacity to activate AMPK in mature adipocytes [39,40]. The mechanism of action by which these compounds exerted this activating action on AMPK could be explained by their direct actuation as agonists of AMPK, linking to the AMP binding sites of the gamma subunit and/or the different sites of the interaction zones between the beta and gamma subunits [40]. Since these results showed the ability of *L. citriodora* compounds to activate the AMPK pathway, this mechanism could be considered a strategy against obesity-related metabolic disorders [39,40].

On the other hand, the effect of verbascoside on mTOR has also been tested showing the ability of this compound to inactivate PI3K/Akt and activate the PI3K/Akt/mTOR signaling pathway in PC12 cells and colorectal cancer cells. [41,42].

The effect of the extract or isolated compounds of *L. citriodora* has also been evaluated by different authors in rabbits and obese/overweight human. In this scenario, verbascoside demonstrated the ability to activate SIRT1 activity in rabbit’s heart and liver. This SIRT1 can regulate antioxidant genes and participates in the regulation of certain signaling pathways, such as AMPK activation, associated with metabolic disorders and obesity. Therefore, the antioxidant effect of verbascoside could be regulated by increasing SIRT1 activity, leading to treatment of obesity and related metabolic disorders through AMPK activation [63]. Herranz-López et al. (2019) conducted a trial in 56 obese/overweight subjects for 2 months in which a dietary supplement containing 500 mg of a combination of phenolic extracts from *L. citriodora* and *H. sabdariffa* (LC-HS) was administered. The consumption of this dietary supplement showed a reduction in symptoms associated with obesity-related diseases. In addition, the LC-HS extract significantly reduced lipid content and increased AMPK activity in a model of hypertrophied adipocyte cells. Therefore, the results of this study suggested that this dietary supplement causes a modulation of fat metabolism in adipose tissue, probably mediated by the activation of AMPK [57].

### 2.3. Hibiscus Sabdariffa

*H. sabdariffa* is known for presenting antioxidant, antigenotoxic and biomodulatory effects in animals exposed to toxic agents. This plant also has diuretic, antimicrobial, and antihypertensive effects [64]. In this sense, Kim et al. (2007) showed that *H. sabdariffa* acts as a mitogen-activated protein kinase (MEK) inhibitor. This inhibition blocks significantly the ability of MEK 1/2 to phosphorylate extracellular signal-regulated kinase 1/2 (ERK1/2) and the expression of PPARγ and C/EBP-α during adipocyte differentiation induction [26].

Moreover, there are numerous studies conducted in different animal models in which *H. sabdariffa* has demonstrated an effect on energy metabolism. For example, phenolic compounds of *H. sabdariffa* inhibited inflammation processes by down-regulating cyclooxygenase-2 (COX-2) and inhibiting the activation of p38 kinase and c-Jun N-terminal kinase (JNK), postulating a relationship between *H. sabdariffa* polyphenols, oxidative stress and suppression of nuclear factor-κB (NF-kB) translocation in a lipopolysaccharide-induced inflammation rat model [47,48].

Regarding, PPARγ and C/EBP-α, when the gene expression of both transcription factors PPARγ and SREBP-1c is observed, the last one is regulated by C/EBP-α [65]. In fact, in obese mice, the supplementation with *H. sabdariffa* extract reduced the transcription of both factors [49]. However, the inactivation of these transcription factors occurs with the activation of the AMPK, for example, by the phenolic compounds present in *H. sabdariffa* extracts. So, these results suggested that the effects of *H. sabdariffa* over adipogenesis are mediated by SREBP-1c and PPARγ regulation through AMPK activation [49].

On the other hand, AMPK and PPARs also participate in the pathogenesis of both alcoholic liver and non-alcoholic fatty liver diseases, in which fatty acid oxidation is impaired. It has been checked that the ameliorating of both diseases can be achieved with the administration of AMPK or PPARα activators both in vitro and in vivo. Phenolics compounds have also demonstrated their effects by increasing PPARγ mediated adiponectin expression, along with AMPK activation [47].

In addition, synergistic effects of *L. citriodora* and *H. sabdariffa* extract, Metabolaid^®^ (MetA), on the metabolism of obese mice fed a high-fat diet were analyzed by Lee et al. (2018). In this study, with the administration of MetA, it was observed that body-weight gain, white adipose tissue (WAT), liver weight, serum, and hepatic lipid profiles and serum glucose levels were reduced [50]. Besides, MetA affected to numerous metabolic pathways more significant than that of with the extracts alone. For example, MetA reduced significantly the expression of the adipogenesis-related genes, CEBP/α, PPARγ, and SREBP-1c and their target genes, aP2/FABP4 and FAS, whereas increased the thermogenesis-related genes as uncoupling protein 1 (UCP1) and uncoupling protein 2 (UCP2) in WAT. Moreover, this extract increased the activation of AMPK and fatty oxidation-related genes, PPARα and ACS1, while the lipogenesis-related genes were decreased [50].

### 2.4. Theobroma Cacao

The action of several phytochemicals of *T. cacao* has been evaluated in different cell lines such as 3T3-L1 preadipocytes, high-glucose treated HepG2 cells or rat Müller cells.

Theobromine is a methylxanthine from the *T. cacao*. This compound has shown to suppress the activation of the Akt/mTOR and NF-κB pathways by in vitro and in vivo models [32,55]. In addition, theobromine has also shown the ability to inhibit adipocyte differentiation in 3T3-L1 preadipocytes through down-regulation of PPARγ, C/EBPα, aP2 and leptin. Specifically, this compound exerts its inhibitory effect at the early stage of adipogenesis through AMPK activation and suppression of the c-Jun N-terminal kinase (JNK) and ERK signaling pathways in 3T3-L1 adipocytes [28].

Regarding flavonoids, epicatechin, a cocoa flavanol, decreases total lipid included in high glucose-exposed hepatic cells via Akt, AMPK, and PKCζ [25]. This compound also reduces the levels of SREBP1-c and FAS and increases the levels of PPARα in high-glucose treated HepG2 cells through the inhibition of PKCζ [26].

Duarte et al. (2015) evaluated the possible protective effects of cocoa on the diabetic retina. In this study, rat Müller cells were treated with cocoa extract enriched in polyphenols. It was observed that the activation of PARP-1 and the decrease of the levels of NAD^+^ and SIRT1 in diabetes were dose-dependent with oral administration of polyphenolic cocoa extract. This study showed that the cocoa extract improved the SIRT1 retinal pathway, allowing the protection of the retina in diabetic patients [43].

The effect of some compounds of *T. cacao* have also been evaluated in different animal models such as hypertensive rats with induced diabetes and obesity rat model induced by a high-fat diet. In this regard, Papadimitriou et al. (2014) evaluated the effect of theobromine treatment in spontaneously hypertensive rats with induced diabetes. They observed that in diabetes mellitus, SIRT1 activity was reduced by PARP-1 activation and NAD^+^ depletion due to low concentration of AMPK increasing NOX4 expression. This is associated with extracellular matrix (ECM) accumulation in the diabetic kidney. Therefore, an increase in the activation of SIRT1 can be achieved by theobromine administration, revealing its therapeutic potential for diabetic nephropathy [51].

Gutiérrez-Salmeán et al. (2014) conducted a study in which epicatechin was administered to obesity rats induced by a high-fat diet. A decrease in the rate of weight gain, blood glucose, and hypertriglyceridemia were detected. In addition, it was observed that epicatechin restored the levels of SIRTs and UCP1 in the skeletal muscle and abdominal tissue of obesity-induce rats [52].

### 2.5. Olea Europaea

Several studies have demonstrated that the fruit and leaf extracts of *O. europaea* possess antihypertensive, antithrombotic, anticancer, antiatherogenic hypoglycemic, anti-inflammatory and antimicrobial properties [29]. These biological activities have been evaluated in different cell models to assess the effects on energy metabolism in human HepG2 hepatocytes, rat liver cells, adventitious vascular fibroblasts, breast cancer cells, SH-SY5Y neuroblastoma cells and 3T3-L1 adipocytes. On this scenario, *O. europaea* fruit pulp extract promotes AMPK phosphorylation in human HepG2 hepatocytes. This phosphorylation causes an activation of AMPK which leads to the inhibition of lipid accumulation due to the activation of low regulation of FAS expression through SREBP-1c inactivation [29].

Concerning main phenolic compounds of extra virgin olive oil, oleuropein, hydroxytyrosol, and tyrosol have been reported to their biochemical and pharmacological properties. The effect of these compounds on the synthesis of lipids in primary cultured rat liver cells has been studied. The results showed the inhibition of the synthesis of *de novo* cholesterol and fatty acids without an effect on the cell viability. In order to clarify the lipid-lowering mechanism of these compounds, the key enzymes in these processes were evaluated, obtaining as a result the reduction of ACC and HMG-CoA reductase activities after 2 h of 25 μM phenol treatment. It was also concluded that the ACC inhibition appears to be mediated by phosphorylation of AMPK [44].

In addition, some secoiridoids from extra virgin olive oil have been reported to activate AMPK, which results in the inhibition of mTOR in breast cancer cells [17]. This inhibition may be important in the action against chronic diseases because in these pathologies there are an overactivation of the nutrient-sensing mTOR due to the loss of responsiveness to active AMPK, a suppressor of mTOR [21].

Hydroxytyrosol has shown a positive regulatory effect on AMPK allowing the prevention of type 2 diabetes brain damage [66]. Wang et al. (2018) studied the hydroxytyrosol effect in autophagy in adventitious vascular fibroblasts, observing that it regulated the expression of the SIRT1 mRNA and protein and that it finally caused the suppression of Akt/mTOR. The authors concluded that hydroxytyrosol regulated the autophagy of adventitious vascular fibroblasts through the suppression of Akt/mTOR mediated by SIRT [45]. Following this positive regulatory effect of hydroxytyrosol on AMPK, other studies have determined in high glucose-induced neuroblastoma SH-SY5Y cell damage an increase of neuroprotection in db/db mice, which showed hyperglycemia and obesity [56]. Moreover, in 3T3-L1 adipocytes, this compound is able to act positively in AMPK and on genes involved in fatty acid oxidation such as PPARα, Cpt1, and PPARγ [46].

Regarding in vivo studies, the effect of *O. europaea* have been evaluated in model 8 prone to senescence accelerated mice (SAMP8) and db/db mice. In this sense, it has been observed that the high intake of phenolic compounds of olive oil, i.e., a high consumption of hydroxytyrosol, positively increased SIRT1 in SAMP8 mice. This positive effect on SIRT1 led to the stimulation of an antioxidant response and a decrease in oxidative stress in the heart of SAMP8 mice [53].

## 3. Effect of Bioactive Compounds on Inflammation Process

Inflammation is a process that is part of the normal repair process when there is damage to the individual and is essential for protection against bacterial and viral infections and harmful environmental agents. When the inflammation persists over time, it becomes chronic inflammation which has been related to several age-related chronic diseases such as Alzheimer’s disease, atherosclerosis, diabetes, cancer, rheumatoid arthritis, among others [67].

Numerous studies have shown that Nrf2 contributes to the anti-inflammatory process by organizing the arrest of inflammatory cells and regulating gene expression through the antioxidant response element (ARE). This signaling pathway allows the regulation of the expression of anti-inflammatory genes and inhibits the progression of inflammation [14]. Nrf2 is a transcription factor that plays an important role in the control of the response to oxidative stress.

Under basal conditions, Nrf2 is locked by kelch-like ECH-associated protein (Keap1) by its two motifs (ETGE and DLG) leading to CUL3-mediated ubiquitination and finally its proteasome degradation. While under oxidative stress conditions, Nrf2 dissociates from Keap1, thus Nrf2 accumulates in the nucleus and activates the ARE-gene battery [68]. One of this ARE-gene is HO-1, upregulated HO-1 transform the heme into CO, bilirubin and free iron. This CO produced acts as an inhibitor of the NF-ĸB pathway which decreases the expression of pro-inflammatory cytokines as interleukin-6 (IL-6) and tumor necrosis factor α (TNF-α), while bilirubin also acts as an antioxidant. Besides, HO-1 inhibits the activation of pro- and anti-inflammatory cytokines as IL-10 [14].

Although Keap1 is the major regulator Nrf2 activation, Nrf2 can be also activated by additional signal transduction pathways, e.g., JNK, p38 MAPK, AMPK or PI3K/Akt, promoting anti-oxidative effects [68]. Both genetic and pharmacological studies propose the existence of a functional cross-talk between Nrf2 and NF-κB pathways. Nrf2 and NF-κB are key pathways involved in controlling the balance of cellular redox status and responses to inflammation and stress [69]. When a situation of oxidative stress occurs, IĸB kinase (IKKβ) is activated raising the release and nuclear translocation of NF-κB. At the nuclear site, NF-κB causes the transcription of pro-inflammatory mediators such as IL-1, IL-6, TNF-α, inducible nitric oxide synthase (iNOS) and intracellular adhesion cyclooxygenase-2 (COX-2) [14]. Excessive increase in IL-1, IL-6 and TNF-α occurs frequently in an acute inflammatory response and in several chronic inflammatory diseases [70].

The activity of NF-κB influences Keapl/Nrf2/ARE signaling pathway in different ways. One of these is Keap1, which degrades IKKβ by ubiquitination, resulting in the inhibition of NF-ĸB activity. Furthermore, inflammatory mediators like COX-2 are produced in the inflammatory processes reacting with Keap1 and activating Nrf2, thereby initiating gene transcription with simultaneous inhibition of NF-κB activity [14].

Numerous in vivo studies have demonstrated that Nrf2 plays an important effect in several inflammatory diseases such as gastritis, arthritis or neurodegenerative disease. In this sense, Ahmed et al. (2017) demonstrated that the absence of Nrf2 reflected greater symptoms of inflammation [14]. Table 2 shows the effects of the selected matrices on the main metabolic pathways related to inflammation process.

### 3.1. Silybum Marianum

Isosilybin, a compound present in *S. marianum* extract presents pharmacological activities which have been reported on in vitro studies. For example, Zhou et al. (2016) reported that isosilybin could alleviate the Aβ _25-35_-induced oxidative stress damage, which is considered a direct cause of Alzheimer’s disease, in HT-22 hippocampal cells. They determined the mechanism of action of isosilybin that significantly increased the protein and mRNA expression of antioxidases, such as HO-1, glutathione S-transferase (GST), and aldo-keto reductases 1C1 and 1C2 (AKR1C2), through the activation of NRF2/ARE signaling. This activation induces inhibition of ROS accumulation and ultimately alleviating Aβ _25-35_-induced oxidative stress damage in HT-22 cells [71].

**Table 2:** Main parameters of the tests that show the effects of different plant species on the main metabolic pathways related to inflammation.

Assay	Model Type	Source	Effective Dose	Parameters	References
In vitro	HT-22 hippocampal cells	Isosilybin (isolated from *S. marianum* extract)	10 µM for 18 h	Activation of NRF2/ARE signaling	[71]
	3T3-L1 hypertrophic adipocytes	*L. citriodora* extract	400 µg/mL for 48 h	NF-κB expression	[38]
	LPS-stimulated macrophage RAW 264.7 cells	Essential oil from *H. sabdariffa*	50, 100 and 200 µg/mL for 24 h	NF-κB and MAPK signaling pathways activation	[70]
	Lymph node carcinoma of the prostate cells	*H. sabdariffa* leaf extracts	0.5 mg/mL for 24 h	Akt/NF-κB/MMP-9 pathway regulation	[72]
	wild-type and Nrf2 KO astrocytes	(−)-epicatechin (isolated from *T. cacao*)	100 μM for 1 h	Nrf2 regulation	[73]
	LPS-stimulated peritoneal murine macrophages	Extra virgin olive oil polyphenolic extract	25 and 50 mg/mL for 18 h	AMPK phosphorylation and NF-κB nuclear translocation	[74]
	Primary astrocytes	Maslinic acid (isolated from *O. europaea*)	1 and 10 μM for 24 h	NF-κB nuclear translocation	[75]
	CD31+/VEGFR-2+ cells	Oleacein and oleuropein (isolated from *O. europaea*)	1, 2, 5 and 10 μM for 3 h	HO-1 and Nrf2 levels	[76]
In vivo	Rats with arsenic treatment	Silybin (isolated from *S. marianum* extract)	75 mg/kg per day for 4 weeks	Nrf2 and NF-κB expressions	[77]
	NASH mice	Silybin (isolated from *S. marianum* extract)	105 mg/kg per day for 8 weeks	NF-κB nuclear translocation	[78]
	Induced gastric injury rats	Silymarin (*S. marianum* extract)	50 mg/kg for 5 days	Nrf2 and NF-κB expressions and pro-inflammatory cytokines levels	[79]
	Zebrafish	Verbascoside (isolated from *L. citriodora* extract)	400 μg/mL per day for 4 days	Nrf2/ARE signaling pathway activation	[80]
	Rats with induced hepatotoxicity	*H. sabdariffa* extract	100 mg/kg/per day for 4 weeks	NF-κB expression and levels of inflammatory mediators	[64]
	TPA-induced mouse skin	Cocoa polyphenols	40 and 200 mg/kg in a dose	MAPKs and NF-kB signaling pathways activation	[81]
	Rats with induced colon carcinogenesis	Cocoa polyphenols	Cocoa-rich diet for 8 weeks	MAPKs and NF-kB signaling pathways activation	[82]
	Obese rats	Cocoa proteins	150 mg/kg per day for 8 weeks	TNF-α protein and mRNA levels	[83]
	Mice with associated colitis	Cocoa	5 and 10% cocoa in the diet for 2 months	Nrf2 levels and COX-2 expression	[84]
	Autoimmune myocarditis rats	Oleuropein (isolated from *O. europaea*)	20 mg/kg per day for 4 weeks	MAPKs and NF-κB pathways regulation	[85]
	Rats with induced renal injury	*O. europaea* leaf extract	100 and 200 mg/kg for 15 days	NF-κB nuclear translocation and Nrf2, HO-1 and NQO-1 expressions	[86]
	Rats with induced testicular damage	*O. europaea* leaf extract	300 mg/kg daily for 5 days	NF-κB nuclear translocation and Nrf2, HO-1 and NQO-1 expressions	[87]
In vitro/In vivo	PC12 cells Sprague–Dawley rats	Verbascoside (isolated from *L. citriodora* extract)	10, 30 and 10 µM for 12 h (in vitro) 25 mg/kg in three doses at 3, 6 and 12 h (in vivo)	HO-1 expression	[41]
	RAW264.7 macrophage cells/Mice	Dp3-Sam (isolated from *H. sabdariffa*)	50-200 mM for 30 min (in vitro) 15 mM/kg for 4 days (in vivo)	MAPK and NF-κB pathways regulation and levels of inflammatory mediators	[88]

Furthermore, the anti-inflammatory effects of certain compounds present in *S. marianum* have been evaluated by in vivo studies. In this sense, silybin has shown a potential anti-inflammatory effect in rats exposed to an arsenic treatment, an environmental and an industrial pollutant [77]. Milton Prabu and Muthumani (2012) observed that the silybin diet supplementation with arsenic treatment causes the expression of Nrf2 mRNA in the kidney tissue of rats to reverse at the level of the control group. In addition, the administration of this compound to the rats without arsenic treatment increases mRNA even above the group control. In this study, the administration of arsenic caused an over-expression of NF-κB in the kidney tissue that could be reduced to baseline levels with the administration of silybin [77].

The administration of silybin in a nonalcoholic steatohepatitis (NASH) mouse model showed that this compound decreased significantly the elevation of p-IKKα/β which is produced by the NASH in liver [78], allowing the blocking of NF-κB.

Other studies revealed that silymarin up-regulated gastric tissue Nrf2 expression, down-regulated gastric tissue NF-κB p65 protein expression, that is necessary to activate the NF-κB [89], and decreased gastric tissue levels of pro-inflammatory cytokines like TNF-α and IL-6 in rats with indomethacin-induced gastric injury [79].

In general, most of the experimental studies suggest that dietary *S. marianum*/silybin activates antioxidant pathways such as Nrf2/HO-1 and downregulates NFκB.

### 3.2. Lippia Citriodora

*L. citriodora* extract has demonstrated the ability to downregulate NF-κB expression in 3T3-L1 hypertrophic adipocytes. In this cell line, the gene expression of some cytokines was measured observing that the plant extract significantly reduced the expression levels of IL-1β,IL-6, TNF-α and CCL2/MCP-1 [38].

Verbascoside has been shown to have an anti-inflammatory effect in in vivo studies using zebrafish or Sprague–Dawley rats. Verbascoside stands out for its anti-inflammatory power, since it exerts a regulation on the Keapl/Nrf2/ARE signaling pathway [41,80,90]. In addition, this phenylpropanoid has been considered an activator of Nrf2 and an inducer of HO-1 expression in both in vitro (PC12 cells) and in vivo (Sprague–Dawley rats) assays through activation of the ERK and PI3K/Akt signal pathways [41]. In another study, the protective capacity of verbascoside in the treatment of Parkinson’s disease was evaluated in a zebrafish model. It was observed that this compound penetrated the blood brain barrier showing a potential therapeutic value in the treatment of Parkinson’s disease since it prevents movement disorders and activates the Nrf2/ARE signaling pathway to attenuate oxidative stress and inflammation [80].

### 3.3. Hibiscus Sabdariffa

The anti-inflammatory effect of *H. sabdariffa* extract has been evaluated in different cell lines, such as macrophages, lymph node carcinoma of prostate cells and macrophage cells RAW264.7; while there are in vivo studies that have evaluated the effect of this matrix in mice and rats with hepatotoxicity.

The anti-inflammatory activity of the essential oil extracted from *H. sabdariffa* was evaluated in LPS-stimulated macrophage RAW 264.7 cells showing an inhibition of the activation of NF-κB and MAPK (JNK and ERK1/2) signaling pathways that resulted in a decrease of NO and pro-inflammatory cytokine (IL-1, IL-6, TNF-α, COX-2, and iNOS) production [70].

Chiu et al. (2015) evaluated the action of *H. sabdariffa* leaf extracts in lymph node carcinoma of the prostate cells resulting in an inhibitory effect on the activity and expressions of matrix metalloproteinase-9 (MMP-9). The MMP-9 inhibition is a consequence of NF-κB inactivation by the *H. sabdariffa* leaf extracts mediated by Akt [72].

Anthocyanins are the major phenolic compounds in *H. sabdariffa*, suggesting that the anti-inflammatory capacity of this plant is caused by these compounds. For example, delphinidin 3-sambubioside (Dp3-Sam) showed an anti-inflammatory effect by in vivo and in vitro models. The administration of Dp3-Sam in mice inhibited the production of inflammatory factors, such as IL-6 and TNF-α, through the downregulation of cellular signaling including MAPK and NF-κB pathways. On the other hand, the levels of inflammatory mediators, such as iNOS, NO, IL-6, MCP-1, and TNF- α induced by lipopolysaccharide in RAW264.7 macrophage cells, decreased after the administration of Dp3-Sam [88].

Moreover, *H. sabdariffa* extract has shown in rats with thioacetamide (TAA)-induced hepatotoxicity the capacity to inhibit the expression of NF-κB and to decrease the levels of TNF-α, IL-6 and IFN-γ, which would indicate a hepatoprotective effect [64].

### 3.4. Theobroma Cacao

The anti-inflammatory effect of the compounds of *T. cacao* have been evaluated in vitro in primary astrocytes isolated from wild-type and Nrf2 KO mice and in vivo studies.

Several studies support the idea of the anti-inflammatory capacity of cocoa phenolics. For instance, the mechanism of action of (−)-epicatechin in this pathway was evaluated in primary astrocytes isolated from wild-type and Nrf2 KO mice. In addition, the results revealed that this compound protected astrocytes against hemoglobin toxicity through the positive regulation of Nrf2 and the inhibition of the activity of activator protein 1 (AP-1). Both cases were mediated by the suppressive action of the compound in the phosphorylation of JNK and by the nuclear expression of JNK, c-jun, and c-fos. Besides that, the inhibitory action of (−)-epicatechin on AP-1 activity was independent of Nrf2, since it occurred in both wild-type and Nrf2 KO astrocytes. Moreover, the positive action of (−)-epicatechin in Nrf2 did not alter the expression of HO-1, which could be the result of a balance between the signaling pathways of Nrf2 activation and AP-1 inhibition [73]. On this scenario, Lan et al. (2017) reported the effect of (−)-epicatechin on the reduction of hemorrhagic stroke injury through the Nrf2 signaling pathway [73].

Cocoa polyphenols have shown the capacity to inhibit the expression activation of MAPKs and NF-kB signaling in 12-O-tetradecanoylphorbol-13-acetate (TPA)-induced mouse skin that cause superoxide-anion generation [81] and in rats model of azoxymethane (AOM)-induced colon carcinogenesis, respectively [82].

Both TNF-α protein and mRNA levels thereof measured in white adipose tissue and the pro-inflammatory chemokine MCP-1 decreased when cocoa protein was administered in rats after a high fat diet [83]. Besides that, the secretion and the mRNA levels of pro-inflammatory cytokines as TNF-α and IL-6 were inhibited by theobromine treatment [28].

Pandurangan et al. (2015) explored the antioxidant properties of cocoa in a mouse model with colitis-associated cancer induced by azoxymethane (AOM)/dextran sulfate sodium (DSS). This study revealed that the administration of 5 and 10% cocoa in the diet caused a higher Nrf2 accumulation in the nucleus of colon cells and reduced the COX-2 expression, an inflammatory mediator that is increased during AOM/DSS-induction [84].

### 3.5. Olea Europaea

The possible anti-inflammatory action of the extract of *O. europaea* or isolated phenolic compounds of this matrix has been evaluated by various authors in both in vitro and in vivo assays. In the case of in vitro assays, peritoneal murine macrophage models stimulated with LPS, astrocytes induced by lipopolysaccharide and different types of cells with induced inflammation have been used.

In this way, Cárdeno et al. (2014) evaluated the potential anti-inflammatory mechanisms of the phenolic extract from EVOO on LPS-stimulated peritoneal murine macrophages. They obtained that this extract decreased LPS-induced inflammatory responses through the reduction of AMPK phosphorylation and the prevention of the NF-κB translocation [74].

Some of the compounds responsible for the anti-inflammatory capacity of the olive leaf and fruit, such as maslinic acid [75], oleuropein [76,91], oleacein [76], hydroxytyrosol and tyrosol [92], have been isolated and tested to verify their effects on the main metabolic pathways involved in the inflammatory response. For example, maslinic acid, an oleanane-type triterpenoid, has shown an anti-inflammatory effect in astrocytes induced by LPS. The results demonstrated that maslinic acid attenuated LPS-induced translocation of NF-κB p65 subunit to the nucleus and avoided LPS-induced IκBα phosphorylation in a concentration-dependent manner [75].

Furthermore, hydroxytyrosol and tyrosol have shown an anti-inflammatory effect in several studies. These compounds were capable of decreasing the NF-κB pathway and downregulating genes and pathways related to inflammation, such as COX-2, IL-1β, TNF-α, Nrf2, HO-1, in different cell lines with induced inflammation [92,93,94]. Another study revealed that both oleuropein and oleacein caused a strongly increased expression of HO-1 and Nrf2 levels in CD31^+^/VEGFR-2^+^ cells decreased by angiotensin II treatment [76]. In addition, the effect of oleuropein was tested in experimental autoimmune myocarditis rats, resulting in a decrease in serum production of TNF-α, IL-1β and IL-6 and an attenuation of the MAPKs and NF-κB pathways [85].

Finally, other parts of the plant besides the fruit also have bioactive compounds that can exert beneficial effects on health. Thus, olive leaf extract has been tested in rats with cyclophosphamide-induced renal injury [86] and in rats with testicular damage induced by intraperitoneal injection [87]. In both models, the inflammation caused by the injury was reduced by the effect of olive leaf extract. This anti-inflammatory response was exerted due to the capacity of the compounds present in the olive leaf extract to inhibit the translocation of NF-κB, suppress pro-inflammatory markers and increase the expression of Nrf2, HO-1 and NQO-1.

## 4. Effect of Bioactive Compounds on Aging

Aging is a complex process characterized by the accumulation of molecular, cellular and organ damage. All these damages lead to loss of function and increase vulnerability to disease and death [95]. The hallmarks of aging can be organized into three key features. The primary hallmarks consist of harm to cellular functions: telomere attrition, genomic instability, loss of proteostasis and epigenetic alterations. Due to these damages, a series of antagonistic responses are produced: deregulated nutrient detection, altered mitochondrial function and cellular senescence. Finally, integrative hallmarks give rise to the characteristic clinical phenotype of the aging that ends in the physiological reserve loss, the decrease of organs and the reduced function [96].

Several studies have shown that dietary or calorie restriction increase the lifespan of diverse organisms, furthermore in monkey rhesus and humans this restriction protects against age-related pathologies like cancer, diabetes, Alzheimer’s disease and cardiovascular disease [95].

The extension of lifespan mediated by the calorie restriction is mediated by mechanisms that end in autophagy. Furthermore, calorie restriction downregulates the mTOR pathway and upregulates SIRT1 deacetylase and the AMPK pathway, all of these pathways also upregulate autophagy [96]. Autophagy decreases with age, improving this degradation route of misfolded proteins, damaged organelles and intracellular pathogens which will prevent their accumulation and, therefore, related diseases. If attention is paid to the effect of metabolic regulation on aging, the downregulation of mTOR pathway, one of the main nutrient sensors and metabolic regulation, either with rapamycin or deletion of ribosomal S6 protein kinase 1 (S6K1) has shown an increase of mouse lifespan [95]. Another important protein in the detection of nutrients is AMPK, this kinase is activated with nutrient starvation, caloric restriction, and exercise, since there is a decrease in ATP and an increase in AMP in these processes. AMPK activation has been reported to promote cell survival under environmental stress and this is related to autophagy [96]. In this sense, a high level of AMP has been also linked to the accumulation of NAD that participates in caloric restriction. High levels of NAD activate sirtuins in situations of nutrient deprivation or growth inhibitory signaling. There is evidence that sirtuins participate in the regulation of insulin signaling and that they act as regulators of longevity [96]. Nevertheless, the role of sirtuins is still unclear.

On the other hand, the mitochondrial free radical theory of aging states that continued exposure to reactive oxygen species causes mitochondrial DNA to increase susceptibility to oxidative damage and aging [97]. Consequently, any damage that affects mitochondrial DNA, dynamics, biogenesis or integrity resulting in deregulated metabolism that is associated with aging.

Another highlight is the ability of different homeostasis maintenance mechanisms in mammals. This responds to different nutrients and hormones to maintain physiological blood glucose levels [96]. With age, many of these sensors and molecular targets are lost or decreased, and this is related to diseases associated with old age, such as cardiovascular and metabolic disorders, muscular atrophy, neurodegenerative diseases and cancer susceptibility [96].

**Table 3:** In vivo studies of the included sources on the main metabolic pathways related to aging.

Model Type	Source	Effective Dose	Parameters	References
Aging mice	*S. marianum* protein hydrolysate	400, 800 and 1.200 mg/kg daily for 7 weeks	Liver mitochondria damage	[98]
*C. elegans*	Silymarin (*S. marianum* extract)	25 and 50 μM	Lifespan	[99]
Rabbits	*L. citriodora* extract	2.2 g per 100 kg of feed	Oxidative damage markers	[100]
Rabbits	Verbascoside (isolated from *L. citriodora*)	5 mg/kg for 80 days	Sirt1 activity and antioxidant levels	[63]
Yeast BY4741	*H. sabdariffa* extract	100 µL of 300 ppm	Lifespan	[101]
Medaka fish	Phenolic compounds-enriched cocoa extract	1, 4 and 8 mg/mL for 4 days	Lifespan and oxidative stress	[102]
Aged rats	*O. europaea* leaf extract	1000 mg/kg daily for 2 months	Antioxidant parameters	[103]
*C. elegans*	Oleuropein aglycone (isolated from *O. europaea*)	-	Aggregation of proteins in amyloid diseases	[104,105]
Type 2 diabetic patients	Silymarin (*S. marianum* extract)	140 mg thrice daily for 45 days	Antioxidant indices	[106]
Elderly individuals	*T. cacao* flavanol consumption	993, 520 and 48 mg daily for 8 weeks	Cardioprotective effect	[107]

Besides, aging is also related to an increase in pro-inflammatory cytokines, which is known to interfere with the insulin action. These cytokines result in both the secretion and production of pro-inflammatory cytokines by increasing the number of senescent cells or by the greater accumulation of visceral fat associated with age, respectively [108]. Certain of these cytokines and inflammatory markers are leptin, TNF-α, IL-6 and plasminogen-1 activation inhibitor. All of them have been related to chronic conditions associated with age [108]. In Table 3, it can be observed different in vivo studies that show the effect of the matrices included in this review on the main metabolic pathways related to aging.

### 4.1. Silybum Marianum

The action of *S. marianum* on metabolic pathways related to aging and its effect on increasing lifespan have been evaluated by different authors using in vivo studies. As an example, d-galactose-induced aging mice were used to prove the antioxidant and anti-aging effects of *S. marianum* protein hydrolysate (SMPH). The results showed that this compound exerted a beneficial effect against aging since it restored the damage induced by d-galactose in liver mitochondria [98]. In this sense, silymarin and 2,3-dehydrosilybin A/B (DHS) from *S. marinanum* have also been shown to promote lifespan in *C. elegans* [99].

On the other hand, it is known that several neurodegenerative diseases related to age occur when there is an increase in oxidative stress, so it is a key aspect to be analyzed. Silymarin supplementation improves antioxidant indices, like the activity of SOD, GPX activity and total antioxidant capacity (TAC) whereas it reduces inflammatory markers in type 2 diabetic patients [106].

### 4.2. Lippia Citriodora

Palazzo et al. (2019) evaluated the effect of *L. citriodora* extract in rabbits, observing that there were improvements in the functions of the liver and kidney in the markers of oxidative damage and in the lipid profile of the animals by the action of the extract. Due to the relationship between the antioxidant effect and caloric restriction with the anti-aging effect, these effects exerted by the extract may be related to protection against aging [100].

Other results that support the anti-aging effect of *L. citriodora* are those obtained by Corbi et al. (2018). This study proved the effect of verbascoside in rabbits, demonstrating a relationship between the compound administration and the SIRT1 activation in the rabbit’s heart and liver tissue. This suggested that SIRT1 could mediate the antioxidant effects of this extract [63]. In addition, the activation of SIRT1 is related to an increase in autophagy, which in turn increases animal’s lifespan [96].

### 4.3. Hibiscus Sabdariffa

The anti-aging effect of *H. sabdariffa* was evaluated by Sarima et al. (2019) observing that the *H. sabdariffa* petal extract extended the lifespan of yeast BY4741 under oxidative stress and normal conditions. The extract was able to induce intracellular mechanisms against oxidative stress such as modification of mitochondrial activity and upregulation of genes involved in tolerance mechanism opposed to oxidative stress and key genes in aging pathway like SIR2 [101]. Moreover, the antigenotoxic effect associated with the hibiscus extracts has been related to its antioxidant capacity and therefore to the anti-aging effect [109,110].

### 4.4. Theobroma Cacao

The effect of compounds present in the *T. cacao* extract on aging has been evaluated in both in vitro and in vivo assays. Phenolic compounds-enriched cocoa extract has also shown to participate in the regulation of the expression of several genes involved in oxidative stress and prolonged lifespan in medaka fish. The extract induced the activation of the antioxidant defense when an oxidative stress situation is produced. Regarding the lifespan extension, in medaka fish, the activation of the SIRT1 protein did not occur directly but was done indirectly in a similar way to that produced in mammals. The extract activated Epac1, a cAMP effector protein, which leads to high levels of CAMP and indirectly activates the enzymatic function SIRT1 [102].

On the other side, one of the diseases with a high prevalence in old aging is cardiovascular disease. Cocoa phenolics have shown cardioprotective effects in different studies that were carried out both in cell and in human trials through the antioxidant, anti-inflammatory and antihypertensive properties of these phenolic compounds from cocoa [107,111,112].

### 4.5. Olea Europaea

Olive phenolic compounds have been linked to an anti-aging effect due to several capacities to produce protective effects. The administration of *O. europaea* leaf extract at a dose of 1000 mg/kg in aged rats showed a decrease in high levels of hepatic malondialdehyde (MDA), diene conjugate (DC) and carbonyl protein (PC) that appears in aged rats. This anti-aging effect presented by the extract may be related to its strong radical scavenging action, acting as an antioxidant itself without affecting the anti-oxidant system [103].

Moreover, protective effects from polyphenols of *O. europaea* have been proven in both *C. elegans* and in humans. Examples of these effects are the interference with the aggregation of proteins in amyloid diseases, the protection of cells and tissues against aging-associated functional disturbance or the transcriptional modulation through epigenetic modifications [104,105]. Olive phenolics have also been linked to anti-aging and cardioprotective properties because they present similar effects to caloric restriction in tissues and organs such as muscle, brain, fatty tissue and kidneys in several ways, but specifically through increased levels and activation of sirtuins [113,114].

## 5. Bioavailability and Pharmacokinetic Properties of Phenolic Compounds

The pharmacological potency of the phenolic compounds is important for the effect that they exert in biological systems. However, their efficacy does not depend only on this potency but also their bioavailability and pharmacokinetic parameters are important. For example, there are phenolic compounds that are rapidly absorbed by the intestinal barrier and plasma in their native state, while others are absorbed in small quantities and may be highly metabolized or rapidly excreted [18]. Therefore, understand the bioavailability, absorption and metabolism of plant polyphenols is essential to determine their mode of action and their final active metabolites.

Accordingly, the metabolites that reach the circulating blood and target tissues may differ from their native forms and the gastrointestinal tract plays a crucial role in this. Phenolic compounds suffer modifications throughout the gastrointestinal tract, first the compounds are hydrolyzed by gastric fluid in the stomach and then metabolized by enzymes of the intestinal cells or catabolized by the microflora of the colon, which can greatly affect the absorption of these compounds [18]. In addition, phenolic compounds can undergo phase I and phase II reactions in the liver. The modifications that the compounds suffer most frequently are glucuronidation, sulfation, and methylation. [11,115].

As mentioned earlier, the five matrices selected to be the subject of this review contain several types of phenolic compounds. As mentioned, whole phenolic extracts or isolated phenolic compounds are responsible for the attributed effect of these matrices on the metabolic pathways related to energy metabolism, inflammation, and aging. However, as indicated in this point, it is important to take into account the metabolites resulting from the ingestion of these matrices on the mentioned metabolic pathways.

In *L. citriodora* verbascoside and isoverbascoside have an effect within the studied metabolic pathways, so it is interesting to consider their bioavailability and pharmacokinetic properties. In this way, the effect of the consumption of an oral acute dose of *L. citriodora* extract (1440 mg/kg) on the antioxidant response of blood cells was further studied in rats using high-resolution mass spectrometry in order to determine other potential metabolites in plasma [116]. In the study, these compounds were found at high levels intact in plasma, suggesting that both compounds could be absorbed in their native forms. However, five other metabolites derived from verbascoside and isoverbascoside by deglycosylation (hydrolysis), methylation or glucuronidation were also found in plasma, namely hydroxytyrosol, ferulic acid, caffeic acid, ferulic acid glucuronide, and homoprotocatechuic acid, together with another eight phenolic compounds [116]. In other study, Wistar rats were orally treated with a dosage of 2000 mg/kg of *L. citriodora* extract that contained a 25% of verbascoside. Blood samples were taken at different times after ingestion and verbascoside was found in plasma samples with a maximum concentration at 20 min. This study concluded that there was a fast absorption of verbascoside in the gut barrier but a very low bioavailability, which may compromise to assign the observed effects to verbascoside. However, there was evidence of the bioactivity of phenylpropanoids at very low concentrations in cell models (at the micromolar range), so low micromolar concentrations of verbascoside over a long-term in plasma could be responsible for some of the effects of the plant [117].

Concerning *H. sabdariffa* polyphenol-enriched extract, the bioavailability and pharmacokinetic parameters were evaluated in Wistar rats after an acute oral dose of 1200 mg/kg [118]. In this study, it was detected in plasma a total of 17 compounds, 11 of these were metabolites. Phenolic acids appeared in plasma without any modification while most of flavonols were found as kaempferol or quercetin glucuronide conjugates, reaching maximum concentration at 120 min. Of all the quercetin and kaempferol derivatives in plasma, both quercetin glucuronide and aglycone had the highest concentrations. These compounds showed higher removal values, revealing an accumulation of tissue and a probable long-term effect [118].

Cocoa polyphenols include a subclass of flavonoids, namely flavan-3-ols, occurring as monomers, mainly epicatechin and catechin, oligomers (procyanidins B1, B2, and C1) and polymers (up to ten units), known as procyanidins [119]. The metabolism of flavan-3-ols has been frequently studied because they have experienced being more bioavailable compared to other cocoa polyphenols. The metabolic changes of frequent flavan-3-ols cocoa have been studied after consumption as well as the physiological levels of epicatechin and catechin [115]. For example, Actis-Goretta et al. (2012) clarified that after the consumption of dark chocolate, (−)-epicatechin-3’-β-d-glucuronide, (−)-epicatechin 3’-sulfate and 3’-*O*-methyl epicatechin sulfates substituted at positions 4′, 5 and 7 were the most relevant metabolites of (−)-epicatechin found in plasma. In addition, the total urine excretion of (−)-epicatechin was 20% of the amount ingested [120].

Taking into account hydroxytyrosol from *O. europaea*, the bioavailability of this simple phenol has been explored in humans, for example in the trial conducted by Miro-Casas et al. where quantified hydroxytyrosol and its main metabolite such as 3-*O*-methylhydroxytyrosol in plasma and urine after a dose of 25 mL of virgin olive oil in healthy humans. The results showed that hydroxytyrosol and 3-*O*-methyl-hydroxytyrosol in plasma increased as a response to virgin olive oil administration, reaching maximum concentrations at 32 and 53 min and that approximately 98% of hydroxytyrosol was present in conjugated forms, mainly glucurono-conjugates. With these results, the authors suggested that ingested hydroxytyrosol can be extensively metabolized for the first time in the intestine and liver and that the biological activity of this compound may be probably derived from its metabolites [121]. In another study, nine healthy volunteers were randomized to receive encapsulated or liquid olive leaf extract as a single lower dose (51.1 mg of oleuropein, 9.7 mg of hydroxytyrosol) or higher (76.6 mg of oleuropein, 14.5 mg of hydroxytyrosol). Then, the opposite dose (but the same formulation) one week later was administered. In the study, fifteen phenolic olive compounds were rapidly found in plasma and urine. They were mainly metabolites derived from phase II; three of them were metabolites derived from hydroxytyrosol, four from oleuropein aglycone and two from homovanilic acid, being hydroxytyrosol glucuronide found in greater quantity. In addition, new metabolites derived from oleuropein in the urine were identified, such as elenolic acid, homovanilic alcohol sulfate and glucuronide of elenolic acid. These results confirmed the rapid absorption of phenolic compounds from the extract of *O. europaea* and the extensive biotransformation of hydroxytyrosol and oleuropein into metabolites, mainly as glucuronidated conjugates, as mentioned above [122].

In the case of the *S. marianum*, no bioavailability and pharmacokinetic studies have been found to comment on the main metabolic transformations of its main phenolic compounds.

**Figure 1:**
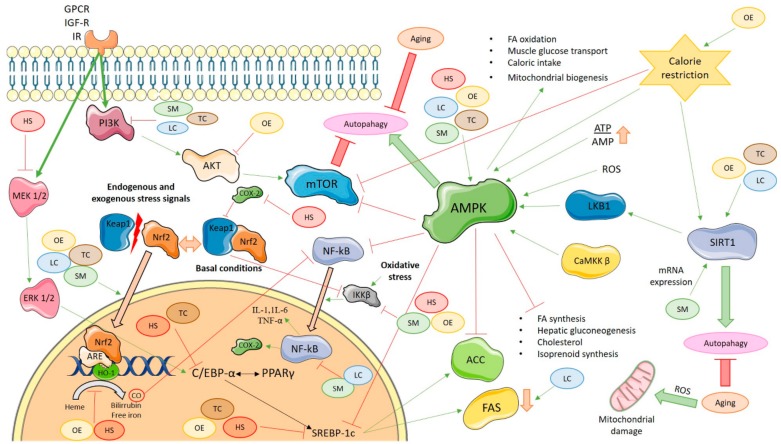
Molecular signaling pathways that are activated or inactivated by dietary phenolics or their metabolites in oxidative stress, inflammation process, and aging. SM: *Silybum marianum*; LC: *Lippia citriodora*; HS: *Hibiscus sabdariffa*; TC: *Theobroma cacao*; OE: *Olea europaea;* AMPK: AMP-activated protein kinase; mTOR: the mammalian target of rapamycin; ATP: Adenosine triphosphate; AMP: Adenosine monophosphate; ROS: Reactive oxygen species; LKB1: liver kinase B1; CaMKK β: Ca2^+^/calmodulin-dependent protein kinase β; SIRT1: Sirtuin 1; ACC: Acetyl-CoA carboxylase; FAS: Fatty acid synthase; NF-kB: Nuclear factor-κB; IKKβ: IĸB kinase; COX-2: Cyclooxygenase-2; Akt: Protein kinase B; PI3K: Fosfoinositol 3-quinasa; Keap1: Kelch-like ECH-associated protein; Nrf2: Nuclear factor-erythroid 2 p45-related factor 2; MEK 1/2: Mitogen-activated protein kinase 1/2; ERK 1/2: Extracellular signal-regulated kinase 1/2; GPCR: G protein-coupled receptor; IGF-R: Insulin-like growth factor receptor; IR: Insulin receptor; ARE: Antioxidant response element; HO-1: Heme oxygenase 1; CO: Carbon monoxide; C/EBP-α: CCAAT/enhancer-binding protein α; PPAR-γ: Proliferator Peroxisome Activated Receptor-γ; SREBP-1c: Sterol regulatory element-binding protein-1c; IL-1: Interleukin-1; IL-6: Interleukin-6; TNF-α: Tumor necrosis factor α. Thin green arrows indicate the activation of a molecule; thin red lines indicate the inactivation of a molecule; thick green arrows indicate the promotion of a process; thick red lines indicate the reduction of a process; thick orange lines indicate the translocation of molecules.

Finally, in this section, some of the transformations suffered by the main phenolic compounds of the matrices under study have been summarized. It can be seen that many of these appear in plasma natively and, therefore, can exert the bioactive effect, but also that many of them undergo modifications that make them transform into new compounds with different bioactive capacities from their native form. These results suggest that the aforementioned effects of the natural sources on metabolic pathways (Figure 1) may be due to native forms of the phenolic compounds or their metabolic transformations.

## 6. Conclusions

Beyond the potential of scavenging free radicals of dietary phenolic compounds, multiple cellular and molecular pathways have been shown to be involved in their roles on different chronic disorders, showing their pleiotropic nature.

As can be seen throughout this review, the metabolic pathways related to energy metabolism, inflammation, and aging are closely related and share central molecules in their regulation. Despite this intimate relationship, there are key molecules in any one of them, which are subject to the regulatory effect of phenolic compounds.

Focusing attention on the metabolic pathways involved in energy metabolism, it can be seen that AMPK exerts an effect as a central regulator, since its activation or inhibition can trigger an effect on other important molecules of energy metabolism. In addition, the activation of AMPK leads to the inhibition of ATP-consuming anabolic pathways, which translates into the inhibition of molecule synthesis processes and the activation of reserve cell degradation. Another important molecule in energy metabolism is mTOR, whose negative regulation is directly related to the activation of AMPK. Several studies, both in vitro and in vivo have shown that entire extracts from natural sources have a positive regulatory effect on AMPK and negative on mTOR, highlighting *L. citriodora* extract or its main compound verbascoside isolated, *S. marianum* extract and secoiridoids from extra virgin olive oil.

In the case of the inflammation-related metabolic pathways, there are two main molecules on which the phenolic compounds have been shown to have a regulatory effect, Nrf2, and NF-κB. Nrf2 plays an important effect in several inflammatory diseases because the absence of Nrf2 in the nucleus has reflected greater symptoms of inflammation while the presence of NF-κB in the nucleus triggers an inflammatory response since it causes the transcription of pro-inflammatory mediators. Both Nrf2 and NF-κB have been shown to be the target of the pleiotropic effect of phenolic compounds in both in vitro and in vivo studies. Some examples noted are the effect of silybin and isosilybin, isolated from *S. marianum* extract, whole *L. citriodora* extract and (−)-epicatechin, isolated from *T. cocoa*.

As for the aging-related metabolic pathways, there are many common intermediaries to the two mentioned above, so many of the aforementioned effects of phenolic compounds on energy metabolism and inflammation would also have an effect on the regulation of aging. Note the central regulator of metabolism, AMPK, and sirtuins. The effect of sirtuins on the regulation of longevity has been studied by various authors although their role on aging today is not entirely clear. Because the metabolic pathways related to aging share many molecules with the other two mentioned, the vast majority of the aforementioned effects of the revised phenolic compounds on energy metabolism and inflammation could also be considered a regulatory effect of aging. Therefore, it should be taken into account that verbascoside (isolated from *L. citriodora*) has shown an effect on certain sirtuins, which makes it potentially effective in aging.

From the data summarized in this review, it is very clear that different pathways are involved to trigger their potential even in different pathologies. However, further studies are still needed to assess the therapeutic and pharmacological potentials of these bioactive compounds due, as mentioned in Section 5, to the pharmacological potency of the phenolic compounds also depend on their bioavailability. For this reason, it is necessary to elucidate if the effects of the studied compounds are effective in their native form or if the metabolic transformations thereof are those that actually exert the bioactive effect.

With this review, it is possible to obtain an integrative image of the existing relationships of the metabolic pathways related to energy metabolism, inflammation, and aging. It also highlights the pleiotropic effect of different types of phenolic compounds on key molecules of these metabolic pathways and their relationship with certain chronic diseases.

## Figures and Tables

**Table 1:** Main parameters of the tests that show the effect of selected plant on the main metabolic pathways related to energy metabolism.

Assay	Model Type	Source	Effective Dose	Parameters	References
In vitro	Huh7.5.1 human hepatoma and Jurkat T cells	Silymarin (*S. marianum* extract)	80 µM for 4, 8 and 24 h	AMPK and mTOR pathways	[13]
	Activated T lymphocytes	Silymarin (*S. marianum* extract)	100 µM for 96 h	Cell cycle and PI3K/Akt/mTOR signaling pathway	[37]
	Hypertrophic adipocytes	Verbascoside (isolated from *L. citriodora* extract) and *L. citriodora* extract	400 μg/mL for 48 h of *L. citriodora* 108 μg/mL for 48 h of Verbascoside	Expression of PPARα, FAS, and AMPK	[38]
	3T3-L1 Hypertrophic adipocytes	29 compounds of the *L. citriodora* extract	200 and 400 µg/mL of the *L. citriodora* fractions for 48 h	AMPK activation	[39]
	3T3-L1 mature adipocytes	*L. citriodora* extract Verbascoside, luteolin-7-diglucuronide and loganic acid isolated from *L. citriodora* extract	100, 200 and 400 µg/mL of the whole extract for 24 h 25, 50 and 100 µg/mL of each isolated compound for 24 h	AMPK activation	[40]
	PC12 cells	Verbascoside (isolated from L. citriodora extract)	30 µM for 1, 3, 6, 12, 24 h	PI3K/Akt/mTOR signaling pathway	[41]
	Colorectal cancer cells	Verbascoside (isolated from L. citriodora extract)	100 µM for 72 h	PI3K/Akt/mTOR signaling pathway	[42]
	3T3-L1 preadipocytes	*H. sabdariffa* extract	250, 500, 1000, 2000 and 5000 µg/mL for 36 h	Adipocyte differentiation	[26]
	3T3-L1 preadipocytes	Theobromine (isolated from *T. cacao*)	50, 100, and 150 μg/mL for 6 days	Adipocyte differentiation	[28]
	Human HepG2 cells	Cocoa flavanol epicatechin (isolated from *T. cacao*)	10 µM for 24 h	Lipid metabolism	[25]
	Rat Müller cells	Cocoa enriched with polyphenols	100 ng/mL, 1 μg/mL and 10 μg/mL for 24 h	Sirtuin pathway	[43]
	Human HepG2 hepatocytes	*O. europaea* fruit pulp extract	10, 20, 40 and 80 μg/mL for 24 h	AMPK and SREBP-1c activation	[29]
	Primary-cultured rat-hepatocytes	Oleuropein, hydroxytyrosol and tyrosol (isolated from *O. europaea*)	10 µM for 24 h of each phenol	Lipid synthesis	[44]
	Breast cancer cells	Secoiridoids from extra virgin olive oil	-	AMPK and mTOR activation	[17]
	Vascular adventitial fibroblasts	Hydroxytyrosol (isolated from *O. europaea*)	200 and 400 µM for 24 h	Autophagy	[45]
	3T3-L1 adipocytes	Hydroxytyrosol (isolated from *O. europaea*)	1.0 and 10 μM for 72 h	AMPK and genes involved in fatty acid oxidation activation	[46]
In vivo	Induced inflammation rats	*H. sabdariffa* extract	10, 20, and 40 mg/kg daily for 5 days	Oxidative stress and NF-kB translocation	[47,48]
	Obese mice	*H. sabdariffa* extract	33 mg of total anthocyanins/kg three times a week for 8 weeks	PPARγ and C/EBP-α transcription	[49]
	Obese mice	MetA (mixture of *L. citriodora* and *H. sabdariffa* extracts)	50 and 100 mg/kg once daily for 8 weeks	Adipogenesis-related genes, oxidation-related genes, lipogenesis-related genes expressions and AMPK activation	[50]
	Diabetic rats	Theobromine (isolated from *T. cacao*)	5 mg/kg daily for 12 weeks	NAD^+^/SIRT1 activity	[51]
	Obese rats	Epicatechin (isolated from *T. cacao*)	1 mg/kg daily for 2 weeks	Levels of skeletal muscle and abdominal tissue SIRTs and UCP1	[52]
	SAMP8 mice	Phenolic compounds of olive oil	-	SIRT1 expression	[53]
In vitro/In vivo	HK2 cells SIRT3 knockout mice	Silybin (isolated from *S. marianum* extract)	50 µM for 24 h (in vitro) 200 mg/kg for 7 days (in vivo)	Mitochondrial function	[54]
	Glioblastoma cells Rats	Theobromine (isolated from *T. cacao*)	10 μM for 72 h (in vitro) 0.05% w/w for 40 days (in vivo)	Akt/mTOR pathway NF-κB pathway	[32,55]
	SH-SY-5Y neuroblastoma cells *db/db* mice	Hydroxytyrosol (isolated from *O. europaea*)	10 µM for 24h (in vitro) 10 and 50 mg/kg per day for 8 weeks (in vivo)	AMPK activation	[56]
	3T3-L1 Hypertrophic adipocytes Obese/overweight subjects	Polyphenols derived from *L. citriodora* and *H. sabdariffa* extracts	500 µg/mL for 72h (in vitro) 500 mg/day for 2 months (in vivo)	AMPK activation	[57]
